# The immunotherapy of Alzheimer's disease

**DOI:** 10.1186/1742-4933-1-2

**Published:** 2004-11-12

**Authors:** Marc E Weksler

**Affiliations:** 1 Department of Medicine, Weill Medical College of Cornell University, 1300 York Avenue, New York, NY 10021, USA

## Abstract

Only a small percentage of patients with Alzheimer's disease benefit from current drug therapy and for only a relatively short time. This is not surprising as the goal of these drugs is to enhance existing cerebral function in Alzheimer patients and not to block the progression of cognitive decline. In contrast, immunotherapy is directed at clearing the neurotoxic amyloid beta peptide from the brain that directly or indirectly leads to cognitive decline in patients with Alzheimer's disease. The single trial of active immunization with the amyloid beta peptide provided suggestive evidence of a reduction in cerebral amyloid plaques and of stabilization in cognitive function of half the patients who developed good antibody responses to the amyloid beta peptide. However, 6% of actively immunized Alzheimer patients developed sterile meningoencephalitis that forced the cessation of the clinical trial. Passive immunotherapy in animal models of Alzheimer's disease has provided similar benefits comparable to those seen with active immunotherapy and has the potential of being effective in the half of Alzheimer's disease patients who do not make a significant anti-amyloid beta peptide antibody response and without inducing T-cell-mediated encephalitis. Published studies of 5 patients with sporadic Alzheimer disease treated with intravenous immunoglobulin containing anti-amyloid beta peptide antibodies showed that amyloid beta peptide was mobilized from the brain and cognitive decline was interrupted. Further studies of passive immunotherapy are urgently required to confirm these observations.

## Introduction

The headline "Minimal benefit is seen in drugs for Alzheimer's disease" summarized an article in the New York Times concerning drug therapy of Alzheimer's disease [1]. The inescapable conclusion was that present drug therapy benefits only a very small percentage of the 4.5 million Americans with Alzheimer's disease patients. This is not surprising as the goal of today's drugs is to enhance existing cerebral function in Alzheimer patients and not to attack the basic causes of their progressive cognitive decline.

In contrast to today's drug therapy for Alzheimer's disease, immunotherapy is directed at the neurotoxic amyloid beta peptide that directly or indirectly leads to cognitive decline in patients with Alzheimer's disease. Most investigators believe that the accumulation of amyloid beta peptide in the brain of elderly adults is not only a hallmark of Alzheimer's disease but is the primary cause of cognitive decline [2]. There is secure evidence from animal studies and preliminary evidence from patients with sporadic Alzheimer disease that immunotherapy can block the accumulation of the neurotoxic amyloid beta peptide in the brain and cognitive decline in patients with Alzheimer's disease. Reducing the level of amyloid beta peptide in the brain can be achieved by decreasing the production of the amyloid beta peptide and/or by increasing its clearance from the brain. Decreased production of amyloid beta peptide would follow the inhibition of the beta and gamma secretases, enzymes that cleave the 40/42 amino acid amyloid beta peptides from the 770 amino acid transmembrane amyloid precursor protein (APP) or the augmentation of the activity of the alpha secretase that lowers amyloid beta peptide production [3,4]. Although inhibitors of beta or gamma secretases are known to suppress amyloid beta peptide generation from APP in cultured cells, the available inhibitors are too toxic for clinical use. Furthermore, it is likely that removal of cerebral amyloid beta peptide will be necessary to reverse the accumulated amyloid beta peptide that is already present at the time when Alzheimer's disease is recognized.

Induction or infusion of anti-amyloid beta peptide antibodies prevents the accumulation of cerebral amyloid beta peptide from the brain of mouse models of familial Alzheimer's disease and may work in humans with sporadic Alzheimer's disease [6,7]. Even more encouraging are the reports that immunotherapy reverses cognitive decline in the mouse models of familial Alzheimer's disease and may act similarly in elderly humans with sporadic Alzheimer's disease [8-10]. This article will review and place in historical perspective the development of immunotherapy, in general, and its application to experimental murine models of familial Alzheimer's disease and to a small number of elderly patients with sporadic Alzheimer's disease so far studied.

## Immunotherapy

Immunity was first recognized by the fact that people who recovered from an infection were often protected from re-infection. Thus, Thucydides wrote of the Athens epidemic of plaque that occurred in 430 BC "the same man was never attacked twice – at least fatally" [11]. Twelve hundred years later, Rhazes wrote that smallpox struck children, rarely late in life, and generally only once [12]. The fact that clinical disease conveyed immunity raised the question whether the induction of a mild smallpox infection might protect individuals from this often fatal disease. Variolization, the inoculation with pustular material from a smallpox patient, was first reported in the 17^th ^century in China to protect from virulent smallpox infection [13] and brought to wider attention in 1714 by Timoni's letter to the Royal Society of London reporting on variolization in Turkey [14]. Despite the clinical efficacy of variolation, the dose of a living, virulent, pathogen was difficult to control, leading, at times, to serious morbidity or mortality.

The next advance in immunotherapy followed the realization that exposure to a related, less virulent, infectious organism such as cowpox, protected individuals from the virulent pathogen, smallpox. In England during the second half of the 18^th ^century the link between the fine complexion (no scars of smallpox) of milkmaids and their exposure to cowpox (vaccinia) was recognized [15]. But it was Edward Jenner's publication in 1798 that proved that inoculation with the coxpox, "vaccination", protected susceptible individuals from small pox [16]. In the years since Jenner's publication, many vaccines have been developed to protect humans and animals against infectious diseases [17]. Most vaccine stimulate an immune response against the infectious microorganisms that cause disease although some vaccines stimulate an immune response to toxins they produce [18]. The primary use of vaccines had been to prevent human disease.

At the end of the 19^th ^century, von Behring and Kitasato discovered that immunity to diphtheria and tetanus was conveyed by serum antibodies against the exotoxins of these bacteria [19]. These investigators, knowing that serum antibodies conveyed immunity, showed that serum anti-diphtheria antibodies that had been induced in one animal could be transferred and, thereby, could cure another animal showing symptoms of the disease. Thus, was born the concept of immunotherapy. The clinical impact of this finding would be great as 50,000 German children died each year of diphtheria at the end of the 19^th ^century. The first successful application of immunotherapy to humans occurred in 1891 when a child suffering from diphtheria was cured by an infusion of horse serum containing anti-diphtheria antibodies. The horse serum containing anti-toxin antibodies could be lifesaving although many patients, receiving large doses of xenogeneic proteins, developed serum sickness resulting from the patients' immune response to the foreign serum. During the first part of the 20^th ^century passive immunotherapy of other infectious diseases including pneumococal pneumonia entered clinical practice but was replaced when antibiotics became available during the second third of the 20^th ^century.

Today, passive immunotherapy is attracting renewed interest as a new means to treat neurodegenerative, infectious, autoimmune, and neoplastic diseases [7,20,21]. Passive immunotherapy has, thanks largely to remarkable progress in monoclonal antibody and recombinant DNA technologies, become one of the hottest fields of therapeutics. Humanized or human monoclonal antibodies have increased the efficacy of passive immunotherapy and eliminated serum sickness following injection of xenogeneic serum. Now passive immunotherapy has been developed for patients with chronic diseases including atherosclerotic, neoplastic, and neurodegenerative diseases.

## Active Immunotherapy of Alzheimer's Disease

Immunotherapy of Alzheimer's disease followed the development of a mouse model of familial Alzheimer's disease. In 1995, an APP-transgenic mouse expressing a mutant, human APP gene isolated from a Swedish family with inherited Alzheimer's disease was developed [22]. These mice develop cerebral diffuse deposits of amyloid peptide and amyloid plaques by middle age and were, therefore, a useful model of familial Alzheimer's disease. Four years later, Schenk and his colleagues reported that repeated immunization of such mice with amyloid beta peptide prevented or reversed accumulation of amyloid deposits in the brain of these mice [6]. Despite the dramatic effects of active immunotherapy on cerebral histopathology in APP-transgenic mice, the key question – did immunotherapy prevent cognitive decline – remained to be answered. Eighteen months later it was reported that the cognitive decline seen in the APP-transgenic mice was blocked by active immunization [23-25]. No toxicity was observed following active immunization of APP-transgenic mice, despite some concern that the administration of the neurotoxic amyloid beta peptide might cause untoward effects [26].

These observations offered great promise for the treatment of patients with familial Alzheimer's disease and, perhaps, elderly patients with sporadic Alzheimer's disease. A clinical trial of active immunization of elderly patients with sporadic Alzheimer's disease was organized and initiated in 2001. However, there were reasons to question whether the benefits seen in a middle-aged mice model of familial Alzheimer's disease could be directly extrapolated to elderly patients with sporadic Alzheimer's disease.

First of all, aging is associated with a decreasing immune response [27]. Thus, active immunization with influenza or tetanus vaccines induces less protective immunity in old than young persons or experimental animals. It was reported that a large percentage of old mice and elderly humans following active immunization with amyloid beta peptide did not generate a robust anti-amyloid beta peptide antibody response [9,28]. Secondly, active immunization of elderly humans and old mice stimulates an increase in autoimmune responses despite the lower immune response to the foreign antigen [29].

The clinical trial of active immunization of patients with Alzheimer's disease with amyloid beta peptide, started in 2001, was stopped a year later after 4 patients in the actively immunized group developed sterile meningoencephalitis [30]. Approximately 6% of the 298 actively immunized Alzheimer's disease patients eventually developed sterile encephalitis. One patient with sporadic Alzheimer's disease died 12 months after the last immunization with amyloid beta peptide from a pulmonary embolus. Post-mortem examination of the brain in this patient revealed CD4+ T cells in a perivascular distribution [31]. While neither the function nor the specificity of the T cells infiltrating the brain was determined, it is possible that the age-associated tendency to generate autoimmune reactions led these patients to generate autoreactive CD4+T cells that entered the brain and contributed to encephalitis.

No comprehensive report of the clinical study of active immunization has yet been published but oral presentations and published results from a subset of actively immunized Alzheimer's disease patients have provided some information [9]. It appears that: (i) all patients with sterile encephalitis had been actively immunized with amyloid beta peptide; (ii) there was no correlation between the level of serum anti-amyloid beta peptide antibodies and risk of sterile encephalitis; (iii) certain patients with sterile encephalitis had no detectable anti-amyloid beta peptide antibodies in serum; (iv) one-half of the elderly patients with sporadic Alzheimer's disease who were immunized with amyloid beta peptide did not generate significant titers of anti-amyloid beta peptide antibodies; and finally, (v) in a subset of patients with sporadic Alzheimer's disease, those patients who generated significant levels of serum anti-amyloid beta peptide antibodies had little or no cognitive decline during the year of observation following active immunotherapy.

In summary, active immunization with amyloid beta peptide in elderly patients with Alzheimer's disease appears to be less effective and more toxic than in the middle-aged APP-transgenic mouse model of familial Alzheimer's disease. These differences may reflect the greater age of the patients with sporadic Alzheimer's disease and the decreased antibody responses to vaccines and the paradoxical increase in autoimmune responses in the elderly.

## Passive Immunotherapy of Alzheimer's Disease

Administration of anti-amyloid beta peptide antibodies would bypass immune senescence and would not be expected to lead to T cell-mediated encephalitis. Furthermore, anti-amyloid beta peptide antibodies not only dissolved aggregates of amyloid beta peptide in vitro but also inhibited aggregated amyloid beta peptide-mediated cytotoxicity in vitro [32]. In vivo, passive immunotherapy of APP-transgenic mice with anti-amyloid beta peptide antibodies prevented or reversed cerebral amyloid deposition depending on whether treatment was begun before or after cerebral amyloid deposition had occurred [7]. It has been reported that passive immunotherapy of APP-transgenic mice prevented age-associated cognitive decline on in the APP-transgenic mice after a 6 week course of treatment with anti-amyloid beta peptide antibodies even before there was any detectable decrease in cerebral amyloid plaque number [33]. Preliminary clinical studies showed that infusing a preparation of human intravenous immunoglobulin (IVIg) containing anti-amyloid beta peptide antibodies into 6 elderly patients with sporadic Alzheimer's disease showed significant cognitive improvement during the 6 months of therapy [10]. Elan announced that a phase 1 clinical study of passive immunotherapy with humanized monoclonal anti-amyloid beta peptide antibody in patients with mild to moderate Alzheimer's disease had been started at the end of 2003 .

The potential benefit of anti-amyloid beta peptide antibodies in humans with sporadic Alzheimer's disease was also inferred from studies of cognitive function in a subset of actively immunized patients with Alzheimer's disease [9]. In these patients, there was a direct correlation between the level of serum anti-amyloid beta peptide antibodies and cognitive function one year after active immunization. Thus, the patients with the highest serum levels of anti-amyloid beta peptide had little or no cognitive decline while cognitive function declined markedly in the nearly 50% of patients who generated little or no detectable serum anti-amyloid beta peptide antibodies after active immunization.

Passive immunotherapy of Alzheimer's disease would require repeated administration of anti-amyloid beta peptide antibodies. For this reason, human anti-amyloid beta peptide antibodies should be used to prevent an immune response to the currently available murine monoclonal immunoglobulins. Several methods are known to obtain human anti-amyloid beta peptide antibodies: (i) purification of specific antibodies from human IVIg; (ii) humanization of murine anti-amyloid beta peptide antibodies by replacing framework portions of the murine anti-amyloid beta peptide antibodies with human framework sequences using recombinant DNA technology [34]; (iii) generation of human monoclonal anti-amyloid beta peptide antibodies in vitro by human immunoglobulin phage library display techniques or in vivo by immunization of mice whose immunoglobulin loci have been replaced by human Ig genes [34].

Human IVIg, purified from human plasma, was initially developed as replacement therapy for immunodeficient patients but IVIg has also been shown to be effective therapy in patients with a variety of autoimmune diseases [35]. Recently, we and others have demonstrated that human serum and IVIg have a significant quantity of human anti-amyloid beta peptide antibodies [36,37]. Such polyclonal human anti-amyloid beta peptide antibody preparations inhibit amyloid beta peptide-induced neurotoxicity in vitro.

There is no evidence that anti-amyloid beta peptide antibodies induce sterile encephalitis, observed following passive immunization of several strains of APP-transgenic mice or in the small number of elderly patients with Alzheimer's disease. However, it may be premature to conclude that passive immunotherapy with anti-amyloid beta peptide antibodies does not induce cerebral pathology. It should be remembered that most of the APP-transgenic mice strains tested do not develop amyloid vascular deposits (congophilic angiopathy) that occurs in elderly patients with Alzheimer's disease. It was reported that infusion of murine anti-amyloid beta peptide monoclonal antibodies specific for the N-terminal epitope of the amyloid beta peptide into a strain of APP-transgenic mice, which develop congophilic angiopathy, cerebral hemorrhage was observed [38]. This untoward effect appears to depend upon the epitope specificity of the anti-amyloid beta peptide antibodies.

## The Choice of Anti-amyloid Beta Peptide Antibodies for Alzheimer's Disease

Pre-clinical data and inferences drawn from immunotherapy in patients with sporadic Alzheimer's disease suggest that passive immunotherapy with anti-amyloid beta peptide antibodies is preferable to active immunotherapy for the treatment of elderly patients with sporadic Alzheimer's disease. However, which anti-amyloid beta peptide antibodies would have greatest therapeutic efficacy and least risk of untoward effects for patients with Alzheimer's disease remains to be determined. However, there is general agreement that anti-amyloid beta peptide antibodies to be administered repeatedly to patients should not stimulate an antibody response to the infused immunoglobulin. There is evidence that humans not only can make immune response to therapeutic antibodies but that such immune responses compromise the action of the therapeutic antibodies [39]. To date, the only human anti-amyloid beta peptide antibodies reported to improve cognitive function in elderly patients with sporadic Alzheimer's disease are those contained in human IVIg [11]. In contrast to preparations containing polyclonal human anti-amyloid beta antibodies, several laboratories have produced humanized anti-amyloid beta peptide antibodies including the Elan preparation now in phase I trial. It has not been reported whether this humanized anti-amyloid beta peptide antibody is active in APP-transgenic mice. The therapeutic efficacy of candidate human anti-amyloid beta peptide antibodies can be compared by measuring their capacity to decrease or reverse cerebral amyloid beta peptide accumulation and cognitive decline in RAG-2-deficient, APP-transgenic mice. We have bred these mice that lack lymphocytes and are incapable of generating an immune response to the human anti-amyloid beta peptide antibodies to test antibodies considered for immunotherapy of Alzheimer's disease.

If the therapeutic benefit of polyclonal human anti-amyloid beta peptide antibodies is confirmed, it remains to be determined which antibody or antibodies are responsible for the therapeutic effect. Whether a single monoclonal antibody will be effective in humans as it has been in APP-transgenic mice is not certain. In some infectious diseases, a single monoclonal antibody has not been less protective than a mixture of several monoclonal antibodies [40]. Thus, while each antibody specificity in polyclonal human anti-amyloid beta peptide antibodies may be at a lower concentration than that of a monoclonal antibody, the synergistic effect of multiple antibody specificities may have advantage.

Monoclonal anti-amyloid beta peptide antibodies are known to differ in their fine specificity: isotype, affinity, as well as epitope, aggregate, and Fc specificity [41]. Whether it will be possible to choose an anti-amyloid beta peptide monoclonal antibody based on these characteristics is far from certain. It is likely that testing in immune deficient APP-transgenic mouse would be performed prior to the treatment of patients with Alzheimer's disease.

Monoclonal anti-amyloid beta peptide have different specificities. There are three major epitopes on the amyloid beta peptide: (i) antibodies to the N-terminal epitope (amino acids 1–6) of the amyloid peptide bind to aggregated amyloid beta peptide in vitro as well as cerebral and vascular deposits in vivo and APP (ii) antibodies specific for the central region (amino acids 15–25) of the amyloid peptide bind to APP but not to aggregated amyloid beta peptide in vitro, amyloid plaques or vascular amyloid deposits (iii) antibodies specific for the C-terminal region have been less well studied but reported to lack a therapeutic effect in APP-transgenic mice. This may be the reason why the N-terminal-specific anti-amyloid beta peptide antibodies but not central region-specific antibodies cause cerebral hemorrhage presumably from vessels with amyloid beta deposits [42].

Anti-amyloid beta peptide antibodies that differ in epitope and Fc specificities, dissolve cerebral amyloid plaques and block cognitive decline in APP-transgenic mice [41]. Anti-amyloid beta peptide antibodies, specific for the N-terminal region of the amyloid peptide, are reported to enter the brain, bind to cerebral amyloid plaques, dissolve the plaque, and mediate Fc-mediated endocytosis followed by catabolism of the amyloid beta peptide within glial cells [7]. However, the Fc-mediated pathway is not the only route to the dissolution of amyloid plaques. Direct application of anti-amyloid beta peptide antibodies that bind to amyloid plaques but do not express the Fc region of the molecule dissolve cerebral amyloid plaques [43]. Furthermore, anti-amyloid peptide antibodies dissolve amyloid plaques in APP-transgenic mice that do not express Fc receptors.

Surprisingly, treatment of APP-transgenic mice with anti-amyloid beta peptide antibodies specific for the central region of the amyloid peptide that do not stain cerebral amyloid plaques ex vivo and were not detectable within the brain also cleared cerebral amyloid peptide and plaques [33]. A novel explanation for the mechanism of action of anti-amyloid beta peptide antibodies that do not enter the brain. The "peripheral sink" hypothesis suggests that cerebral amyloid beta peptide in all its forms (monomer, oligomer, fibrils) are in equilibrium with amyloid beta peptide in the blood and, that anti-amyloid peptide antibodies, which cannot cross the blood-brain barrier, deplete cerebral amyloid beta peptide by its mobilization into the blood. It was shown that within a few hours of administering central region-specific anti-amyloid beta peptide antibodies, which do not enter the brain, the level of amyloid beta peptide in the blood increases as much as 1000 fold. Furthermore, the magnitude of the increase in total amyloid beta peptide levels in the blood following a single injection of anti-amyloid beta peptide antibody is a surrogate marker of cerebral amyloid beta peptide load [44]. Thus, it appears that similar effects – decreased cerebral amyloid load and cognitive loss can occur following treatment of APP-transgenic mice with different anti-amyloid beta peptide monoclonal antibodies by central (entry into the brain) or peripheral (entry into the blood) mechanisms.

Both N-terminal-specific and central region-specific anti-amyloid beta peptide antibodies but not C-terminal-specific anti-amyloid beta peptide antibodies also bind to APP the ubiquitous transmembrane cellular protein. C-terminal-specific anti-amyloid beta peptide antibodies can distinguish between amyloid beta 1–40 and 1–42 peptide. As amyloid beta 1–42 peptide is more neurotoxic peptide and forms the nidus of cerebral amyloid plaques antibodies to this amyloid beta peptide might be the most effective antibody for passive immunotherapy as they target the most pathogenic form of the amyloid beta peptide without binding to APP or the less pathogenic amyloid beta 1–40 peptide. However, the C-terminal specific anti-amyloid beta peptide antibodies have been reported not to clear cerebral amyloid beta peptide [41]. Recently, it has been reported that a C-terminal specific anti-amyloid beta peptide antibody does clear amyloid plaques [45].

Finally, there is considerable interest in the greater neurotoxicity of soluble amyloid beta peptide oligomers than either the amyloid beta monomers or fibrils [46]. If this proves to be case, it would be important to test the therapeutic efficacy of monoclonal antibodies to amyloid beta peptide oligomers in APP-transgenic mice [47].

## Conclusion

Passive immunotherapy of sporadic Alzheimer's disease offers the potential of reversing the pathologic accumulation of cerebral amyloid beta peptide. To date the only preparation of human anti-amyloid beta peptide antibodies that have been reported to reverse cognitive defects in patients with sporadic Alzheimer's disease are polyclonal anti-amyloid beta peptide antibodies contained in human IVIg. Selection of human anti-amyloid beta peptide antibodies for clinical trial can be tested for therapeutic effect in vivo by their treatment of immunodeficient APP-transgenic mice.

## Competing Interests

The authors declare that they have no competing interests.
